# Validating a robust double‐quantum‐filtered ^1^H MRS lactate measurement method in high‐grade brain tumours

**DOI:** 10.1002/nbm.3587

**Published:** 2016-08-11

**Authors:** G.S. Payne, L.M. Harris, G.S. Cairns, C. Messiou, N.M. deSouza, A. Macdonald, F. Saran, M.O. Leach

**Affiliations:** ^1^ MRI Unit, Royal Marsden HospitalSutton, SurreyUK

**Keywords:** brain, double‐quantum filter, glioblastoma, lactate, MRS, single voxel

## Abstract

^1^H MRS measurements of lactate are often confounded by overlapping lipid signals. Double‐quantum (DQ) filtering eliminates lipid signals and permits single‐shot measurements, which avoid subtraction artefacts in moving tissues. This study evaluated a single‐voxel‐localized DQ filtering method qualitatively and quantitatively for measuring lactate concentrations in the presence of lipid, using high‐grade brain tumours in which the results could be compared with standard acquisition as a reference.

Paired standard acquisition and DQ‐filtered ^1^H MR spectra were acquired at 3T from patients receiving treatment for glioblastoma, using fLASER (localization by adiabatic selective refocusing using frequency offset corrected inversion pulses) single‐voxel localization. Data were acquired from 2 × 2 × 2 cm^3^ voxels, with a repetition time of 1 s and 128 averages (standard acquisition) or 256 averages (DQ‐filtered acquisition), requiring 2.15 and 4.3 min respectively.

Of 37 evaluated data pairs, 20 cases (54%) had measureable lactate (fitted Cramér–Rao lower bounds ≤ 20%) in either the DQ‐filtered or the standard acquisition spectra. The measured DQ‐filtered lactate signal was consistently downfield of lipid (1.33 ± 0.03 ppm vs 1.22 ± 0.08 ppm; *p* = 0.002), showing that it was not caused by lipid breakthrough, and that it matched the lactate signal seen in standard measurements (1.36 ± 0.02 ppm). In the absence of lipid, similar lactate concentrations were measured by the two methods (mean ratio DQ filtered/standard acquisition = 1.10 ± 0.21). In 7/20 cases with measurable lactate, signal was not measureable in the standard acquisition owing to lipid overlap but was quantified in the DQ‐filtered acquisition. Conversely, lactate was undetected in seven DQ‐filtered acquisitions but visible using the standard acquisition.

In conclusion, the DQ filtering method has proven robust in eliminating lipid and permits uncontaminated measurement of lactate. This is important validation prior to use in tissues outside the brain, which contain large amounts of lipid and which are often susceptible to motion.

## 1 Introduction

Tissue concentrations of lactate are elevated in many tumours, including brain,^1^, ^2^ prostate^3^ and squamous cell carcinoma,^4^ and in stroke,^5^ multiple sclerosis^6^ and inflammation.^7^ Lactate has also been identified as a valuable marker of action in a number of targeted therapeutics and cellular processes.^8^, ^9^, ^10^ However in many tissues measurement of lactate concentration using ^1^H MRS is hampered by the overlap with large lipid resonances.

Several strategies have been explored to improve the discrimination of the lactate resonance at 1.33 ppm in the presence of lipid. In spin‐echo sequences using an echo time of 144 ms, *J* coupling causes the lactate doublet to be inverted relative to singlets and to lipid. This helps identify whether a signal observed at 1.3 ppm is predominantly lactate or lipid, but cannot avoid cancellation between positive lipid and inverted lactate signals when both are present. The use of longer echo times in the acquisition sequence increases the relative size of the lactate peak owing to the greater transverse relaxation rates of the lipid resonance.^11^ However this method is only sufficient when the relative lipid content is small. Another strategy uses spin‐echo difference methods.^12^, ^13^, ^14^, ^15^ These methods rely on the 180° phase difference between lactate signals acquired using a non‐selective RF refocusing pulse, and those acquired using frequency selective refocusing in which the phase of the coupled quartet at 4.1 ppm is not inverted; subtraction of the two data sets eliminates the lipid signals but retains the lactate. This method has the great advantage of enabling the full signal of the lactate to be acquired. Measurement of signals from other metabolites is also possible using the same acquired data, by addition rather than subtraction of the two paired spectra. However, spin‐echo difference methods have a serious disadvantage in the presence of motion, where slight differences in the lipid peak resonances between measurements lead to imperfect lipid signal cancellation. What is required is a single‐shot method that avoids the need for signal subtraction or phase cycles. In this case the use of double‐quantum (DQ) filters^16^, ^17^ is advantageous, as these use magnetic field gradients to spoil the lipid signals, do not rely on subtraction and therefore should be relatively robust to the presence of motion. The penalty for good signal integrity is a reduction in signal amplitude; although DQ filtering schemes that retain the full lactate signal (except for relaxation losses) have been demonstrated, this is only possible for unlocalized acquisitions or for localized acquisitions using a phase cycle. Incorporation of the DQ filter into a single‐shot localization scheme (as required for target tissues experiencing motion) restricts the maximum passed lactate signals to 50%.

We have previously described^18^ a single‐shot DQ‐filtered lactate detection sequence, localized using a semi‐LASER sequence^19^ incorporating FOCI RF pulses^20^ to reduce the chemical shift displacement artefact to a negligible proportion. In test objects the lactate detection efficiency was shown to be about 47%, and the lipid suppression to be better than 99%. Examples of *in vivo* spectra were shown from brain, cervix and breast. Before implementing the method in clinical trials in extracranial tumours it is important to be able to confirm two points. One is that the lipid suppression works as well *in vivo* as it does in the test object, that is, to confirm that the peak detected is indeed primarily due to lactate without significant lipid contamination. The second point is to confirm that the method produces quantitatively correct results for lactate concentration in a clinical context. To address both these points requires measurement in a system in which the lactate can also be detected using a conventional acquisition.

This study therefore evaluated the single‐shot DQ filtering method in a clinical cohort in which lactate is often present and sometimes directly measureable (i.e. without the requirement for a DQ filter). For this a cohort of patients being treated for glioblastoma was selected. In particular, the aims were to demonstrate a consistent frequency shift between measured DQ‐filtered lactate signal and lipid (to confirm that the lactate signal detected does not arise from residual lipid breakthrough), to compare lactate concentrations when they were detectable directly as well as when using the DQ filter, and to verify the operation and utility of the method for routine measurements in patients.

## 2 Methods

The study was performed in a cohort of 20 patients being treated for glioblastoma, with scans acquired at various times during treatment. Patients gave written informed consent, and the study was approved by the local research ethics committee. As the purpose of this technical study was to compare methods for lactate detection rather than looking for changes in relation to treatment, all patient measurements were grouped as one cohort.

Scans were performed on a Philips 3T Achieva TX system (Best, The Netherlands), using an eight‐channel SENSE head coil. They were appended to scans acquired for clinical purposes. These clinical scans were used to guide voxel positioning to the region anticipated to include active tumour. Matched DQ‐filtered and unfiltered MRS data were acquired in the same examination with the same receiver gain. A scan was also acquired without water suppression to evaluate shim linewidths, and as a concentration reference.

For optimal data quality and minimal data collection time, a single‐voxel acquisition strategy was used. To avoid the well‐known partial voxel problem for detection of lipid using the PRESS sequence^21^, ^22^ paired unfiltered and DQ‐filtered spectra were acquired using the fLASER (localization by adiabatic selective refocusing (LASER) using frequency offset corrected (FOCI) inversion pulses) single‐voxel localization method.^18^ This sequence is a modified semi‐LASER sequence,^19^ in which the slice‐selective adiabatic hyperbolic secant inversion pulses are replaced by FOCI RF and gradient pulses,^20^, ^23^, ^24^ in order to achieve high bandwidth (12 kHz) refocusing (and therefore greatly reduced chemical shift displacement artefacts) while using the modest *B*_1_ amplitude available on our body RF transmit coil (13.5 μT). It has previously been demonstrated to suppress lipid signals to less than 1%, while passing about 47% of lactate signals.^18^

All data were acquired from a (2 cm)^3^ voxel, with 2048 complex sampling points over a 4 kHz acquisition bandwidth, and *T*_R_/*T*_E_ = 1000/144 ms. 128 averages were acquired for the unfiltered metabolite spectrum, 256 averages for the DQ‐filtered spectrum, and four averages for the water reference. The Philips ‘frequency stabilization’ option corrects for any effects of frequency drift during the examination (see for example Reference 25) by adjusting the scanner frequency based on measuring the water frequency prior to each localized acquisition. The total acquisition time for all three spectra was about 7 min.

As the DQ‐filtered spectra had low SNR, it was important to minimize the likelihood of attributing noise components or other features to lactate, especially as other metabolites are largely eliminated by the DQ filter and there is no signal that can be used as a reference for phasing the spectra. Signals were only identified as lactate if they were in the expected frequency range (approximately 1.3–1.4 ppm). In addition, spectra containing unidentified signals that could potentially interfere with measurement of the lactate resonance were discarded. Standard acquisition (unfiltered) spectra were also discarded if they showed evidence of outer volume ghosting.^26^ Data were fitted using the Amares algorithm in the jMRUI processing package.^27^, ^28^ In the unfiltered metabolite spectra the main resonances of choline (3.25 ppm), creatine (3 ppm), N‐acetyl aspartate (NAA, 2 ppm) and lipid (1.3 ppm, when present) were modelled as single Lorentzians. Lactate was modelled in all cases as a pair of Lorentzian peaks of equal amplitude and linewidth with a separation of 6.5 Hz, and with a 180° phase inversion relative to the other peaks in the unfiltered data. No attempt was made to fit coupled resonances passing through the DQ filter except for those of lactate.

To partially compensate for low signal in some cases and to avoid widely ranging linewidth estimates, the lactate linewidth was constrained based on the measured linewidth of the water resonance. Peak linewidths are determined primarily by a contribution from magnetic field inhomogeneity (which is common to all peaks in the same voxel), and by their transverse relaxation time constant, *T*_2_. In general brain water has a shorter *T*_2_ (80 ms; Reference 29) than lactate (453 ms; Reference 30), giving corresponding intrinsic peak linewidths of 4.0 Hz and 0.7 Hz respectively. In this case lactate would be expected to be 3.3 Hz narrower than the water resonance. However in many cases the measured water linewidth, LW_water_, in the tumour voxels was found to be less than 4.0 Hz, indicating that these tumours have a longer *T*_2_ than is characteristic of normal brain. In these cases it may be assumed that the lactate linewidth will be less than 3.3 Hz narrower than the water peak, but that the lactate linewidth will not exceed that of water. The lactate linewidth was therefore constrained to lie between (LW_water_ – 3.3) and LW_water_. A peak was regarded as measurable if the Cramér–Rao lower bound (CRLB) was less than 20% of the estimated amplitude.

For comparison of methods of lactate detection, measurement of peak areas in jMRUI is sufficient. Estimating real concentrations from these peak areas is confounded by the uncertainly in the estimates of water *T*_2_ alluded to above, and more particularly by uncertainty in the lactate *T*_2_. The *T*_2_ of lactate is difficult to measure owing both to low concentrations leading to low signal‐to‐noise ratio in the measurements, and to the effects of *j* coupling on the peak shape at different echo times. These factors will partly account for the wide range of values for lactate *T*_2_ reported in the literature (280 – 1200 ms; References 31, 32, 33, 34). In this manuscript indicative estimates of lactate concentration were calculated assuming *T*_1_ and *T*_2_ values for water of 1330 and 80 ms respectively,^29^ a *T*_1_ value for lactate of 1730 ms,^35^ a compromise *T*_2_ value for lactate of 453 ms,^30^ and a brain water content of 41 M (Reference 2). Lactate concentrations calculated from the DQ‐filtered spectra were also corrected for the lactate detection efficiency of the filter (47%; Reference 18). Peak areas for the other metabolites were corrected using *T*_1_ values of 1.06 s, 1.38 s and 1.38 s for choline, creatine and NAA respectively,^36^ and average *T*_2_ values from four publications of 235 ms, 159 ms and 262 ms respectively.^36^, ^37^, ^38^, ^39^ These calculated concentrations will also only be approximate values, as it was not possible to measure the *T*_1_ and *T*_2_ in each case. In addition some scans were performed after contrast injection as part of the clinical protocol. However while this may affect the concentration estimates, it should only have a small effect on the comparison between DQ‐filtered and standard acquisition, as they were acquired one immediately after the other, and at the end of the clinical study, when the contrast agent concentration is not changing rapidly.

Results are quoted as mean ± standard deviation. Comparisons between groups were evaluated using an unpaired *T* test.

## 3 Results

**Figure 1:**
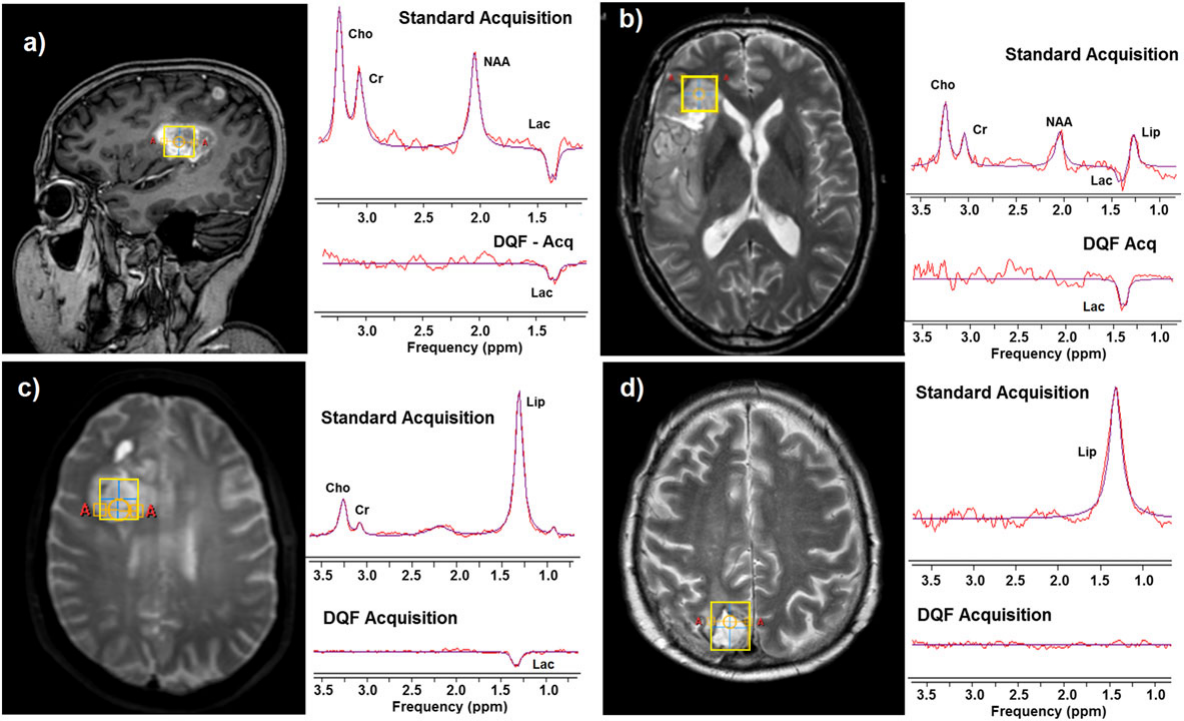
Examples of standard acquisition and DQ‐filtered single‐voxel fLASER spectra in glioblastoma. Voxel location is shown on the left, with standard acquisition and DQ‐filtered acquisition on the right, plotted in each case on the same vertical scale. The smooth curve is the Amares fit to the data. All spectra were acquired at 3T from a cubic voxel with 2 cm sides, 2048 complex sampling points over a 4 kHz acquisition bandwidth, and *T*_R_/*T*_E_ = 1000/144 ms. 128 averages were acquired for the unfiltered spectrum, and 256 averages for the DQ‐filtered spectrum. A, Example with no apparent lipid overlying the lactate. B, Example showing significant overlap between lactate and lipid, with consequent partial signal cancellation. C, Example showing a dominant lipid peak in the standard acquisition but lactate detected in the DQ‐filtered spectrum. D, Example of a dominant lipid resonance in which no lactate was present (below detection threshold in the DQ‐filtered spectrum). Only in A can a reasonable estimate of lactate concentration be obtained from the standard acquisition spectrum.

Examples of spectra acquired using the fLASER sequence are shown in Figure 1, illustrating situations with different relative contributions of lactate and lipid. In each example the standard acquisition (unfiltered) and DQ‐filtered spectra are shown on the same vertical scale. In the example shown in Figure 1A there is negligible lipid, and lactate can be measured adequately using the standard technique. The standard acquisition spectrum of Figure 1B contains more lipid, which partly overlaps with the lactate, while the DQ‐filtered spectrum shows a much larger lactate peak. These data are consistent with the signals of opposite phase in the standard acquisition leading to partial signal cancellation, which is very difficult for any algorithm to fit correctly, while for the DQ‐filtered spectrum the real size of the lactate peak is revealed. In Figure 1C the lipid totally dominates the standard acquisition spectrum, but is eliminated by the DQ filter to reveal measureable lactate. The standard acquisition of Figure 1D looks similar to that of Figure 1C, but the DQ‐filtered spectrum shows that in this case there is no significant lactate beneath the lipid peak. The number of spectra in each group, with their median water linewidths, were 12 (5.9 Hz), 6 (6.2 Hz), 5 (9.7 Hz) and 5 (7.8 Hz) respectively, and nine spectra showed no lactate or lipid (9.3 Hz).

In the unfiltered spectra the measured lactate resonance was consistently downfield of the lipid, with an average lactate frequency of 1.36 ± 0.02 ppm (mean ± sd, relative to water set to 4.67 ppm), compared with 1.22 ± 0.08 ppm for lipid (*P* < 0.001, using a *t* test). The lactate in the DQ‐filtered spectra was also consistently downfield of the lipid resonance (1.33 ± 0.03 ppm; *P* < 0.002), but very similar to the unfiltered lactate frequency. The large difference in frequency of the DQ lactate resonance compared with the standard acquisition lipid resonance, and the close agreement in lactate resonance frequency between the two acquisition methods, increases confidence that the peak measured in the DQ‐filtered spectra is indeed lactate and is not substantially compromised by breakthrough of lipid.

Fifty matched pairs of unfiltered and DQ‐filtered fLASER single‐voxel ^1^H MR spectra were acquired in tumour at various stages of treatment. One pair was discarded owing to the clear presence of ghosting artefact in the standard acquisition spectrum, and a further 12 were left out of the analysis owing to concerns with unidentified signals in the spectrum that could potentially affect lactate quantification. Of the remaining 37 paired data sets, the median shimmed water linewidth was measured as 7.0 Hz (range 3.0–19.3 Hz). Lactate was measureable (defined as CRLB of the fit < 20%) in one or both spectra in 20 cases (54%). Of these, 14/20 had significant lactate in the unfiltered spectra, 12/20 had significant lactate in the DQ‐filtered spectra and 7/20 were measureable in both spectra.

**Figure 2:**
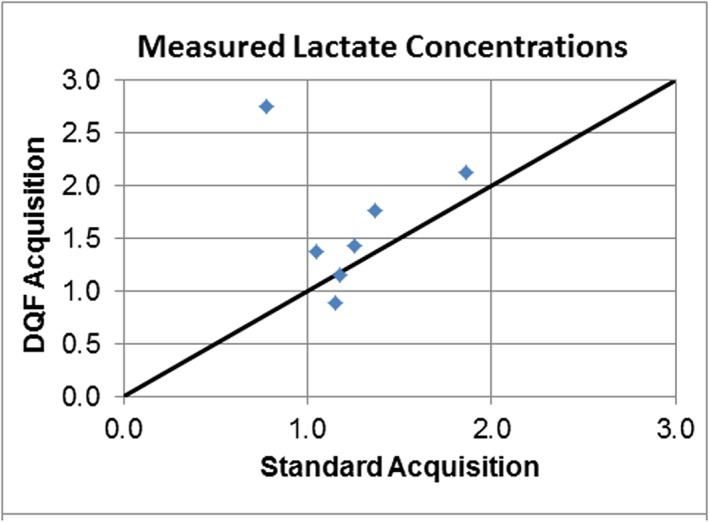
Comparison of lactate concentrations measured using standard acquisition and DQ‐filtered acquisition. The line of identity is indicated. The two measurements yield similar results, except for the outlier with the DQ‐filtered concentration of 2.75 mM. This corresponds to the case shown in Figure 1B, in which the lactate is strongly overlapped with lipid.

For the cases in which lactate was measureable in both spectra the calculated concentrations were generally similar (Table 1, Figure 2). The exception is Case 4, in which the calculated lactate concentration from the DQ‐filtered spectrum is about 3.5 times larger than that from the standard acquisition. This case is that shown in Figure 1B, from which the reason for the discrepancy is clear: i.e., in the standard acquisition the lactate signal is reduced by cancellation from the lipid signal. If this case is omitted the average ratio of DQ‐filtered to standard lactate concentrations was 1.10 ± 0.21, with mean lactate concentrations of 1.46 ± 0.44 mM (DQ filtered) versus 1.31 ± 0.29 mM (standard acquisition). This demonstrates that in the absence of lipid contamination the DQ‐filtered method yields a similar lactate concentration to that from the standard acquisition, and in the presence of lipid it provides a much superior estimate of lactate content. For comparison the average concentrations of the other metabolites were calculated from the standard acquisition spectra to be 1.8 ± 0.9 mM for choline, 4.2 ± 2.6 mM for creatine and 3.2 ± 2.2 mM for NAA.

**Table 1:** Comparison of lactate measurements using standard acquisition or DQ‐filtered acquisition in patients in which lactate was quantifiable using both methods. The lactate frequency is referenced to water at 4.68 ppm. The concentration calculations have been corrected for *T*_1_ and *T*_2_ effects as described in the text

**Data set**	**Water linewidth (Hz)**	**Standard acquisition lactate**		**DQ filtered lactate**	
		**Frequency (ppm)**	**Conc. (mM)**	**Frequency (ppm)**	**Conc. (mM)**
1	6.69	1.344	1.05	1.342	1.38
2	4.45	1.345	1.16	1.283	0.88
3	3.00	1.310	1.86	1.356	2.12
4*	4.85	1.395	0.78	1.348	2.75
5	5.04	1.385	1.26	1.349	1.43
6	3.94	1.363	1.18	1.354	1.16
7	5.82	1.387	1.37	1.375	1.77

* Data Set 4 contained high lipid (Figure 1B), and therefore yielded very low lactate in the standard acquisition.

## 4 Discussion and conclusions

From a technical point of view in order for the measurement of lactate using ^1^H MRS to be used as an imaging biomarker it needs to be demonstrated to be precise, accurate and widely available.^40^ The main purpose of this study was to provide evidence that the DQ‐filtered method gives similar (i.e. relatively accurate) lactate concentrations to those acquired using standard acquisition methods, without contamination from overlapping lipid. This is an essential step towards demonstrating that this single‐shot method is suitable for the measurement of lactate in tissues that experience motion, as well as in relatively static tissues such as brain. The results also give some indication of precision. The method uses standard hardware and so in principle can be implemented on any clinical MR system.

Regarding the accuracy of the measurement, it was found first that the frequency of the peak identified as lactate was the same in both DQ‐filtered and unfiltered spectra, but was shifted relative to the lipid signals. Second, in cases in which lactate is measured using both methods the resulting estimated concentrations are similar, with no sign of much larger DQ‐filtered signals when more lipid was present. This also demonstrates that the estimated lactate detection efficiency (47%) measured previously in a phantom^18^ has not changed significantly for *in vivo* measurements.

While the two acquisition methods produced similar values for relative lactate content, the calculated values for lactate concentrations must be used with some caution, owing to the uncertainty in values of *T*_1_ and *T*_2_. This is a particular problem for *T*_2_, since it is harder to measure and so the literature offers a correspondingly large range of possibilities. In addition, both *T*_1_ and *T*_2_ are likely to change with pathology. Ideally the acquisition parameters would be altered to minimize dependence on *T*_1_ and *T*_2_. This is certainly possible for *T*_1_, by increasing the *T*_R_ from the 1 s used in this study (which was constrained to match a parallel MRSI study, for which a longer *T*_R_ would have resulted in an unacceptably long scan time). A longer *T*_R_ of 2 s would increase the signal‐to‐noise ratio per unit time (assuming the *T*_1_ of lactate is approximately 1730 ms; Reference 35) and also reduce the dependence of estimated lactate concentration on the precise value used for *T*_1_ corrections. However, *T*_2_ effects are determined by the echo time (144 ms), which cannot be changed in this sequence owing to the requirement to match the evolution of the spins under *j* coupling. The uncertainty in the reference measurement associated with the *T*_1_ and *T*_2_ of water can be overcome by using long *T*_R_ to eliminate *T*_1_ effects, and measurement at two echo times to measure the actual water *T*_2_ in each patient.

While the DQ filtering method is not optimal for measurements in brain, in which motion effects are generally small, it clearly can be used for this purpose. Since in many of these brain lesions lactate was detectable using the unfiltered method, a good strategy for future studies in tumours of this type would be to acquire the quicker unfiltered spectrum first, and to proceed with the DQ‐filtered spectrum only if there is a large lipid peak present that potentially obscures or overlaps with lactate.

In this study lactate was measureable (CRLB < 20%) using either the standard acquisition or the DQ filtering method in about half of the cases; the absence of significant lactate in the others implies that the concentration in the selected voxels was below the threshold for detection for the sequence parameters used (approximately 0.5 mM; Reference 18). In this particular cohort, when lactate was measureable lipid concentrations were often low and the lactate could be measured using both methods. However when significant amounts of lipid were present only the DQ filtering method was able to make a reasonable estimate of lactate concentration. In tissues outside the brain, for which the sequence was primarily developed, it is expected that most voxels will contain large amounts of lipid and therefore the DQ‐filtered method will be essential in most cases if a reasonable estimate of lactate concentration is to be made.

In these measurements the unfiltered fLASER sequence used 128 averages, while the DQ‐filtered data were acquired with 256 averages, requiring twice the acquisition time. This increase in scanning time was partly to offset the known reduction in SNR using the DQ filter. While this means that the two methods are not directly comparable from a timing perspective, in general the lack of lactate detection in the unfiltered spectra appeared to be more due to lipid contamination than to low SNR (although this has not been robustly demonstrated).

The DQ filtering method employed can detect a maximum sensitivity of 50% relative to the unfiltered measurement in theory (and 47% in practice). It would be good to acquire the full 100% of the lactate signal, and indeed unlocalized sequences have been demonstrated to do this.^16^ However, it does not seem possible to translate this into a localized sequence while still being single shot (i.e. to avoid the use of a phase cycle). This is because, for singlets (such as water), the insertion of an additional gradient in the first half of the DQ‐filtered period to encode the DQ to ZQ coherence pathway then adds to the existing gradient that encodes the ZQ to DQ pathway (the first blue pulse in Figure 1 of Reference 18), which then permits rephasing by the subsequent double‐length gradient; the spoiler gradients (green in Figure 1 of Reference 18) are no longer effective.

These measurements all used the fLASER sequence. It is well known that conventional single‐voxel localization sequences such as PRESS all suffer from a large chemical shift displacement artefact, caused by the limited available RF amplitude limiting the available bandwidth for the refocusing pulses used for localization. For lactate this produces signals of different phase and shape from different regions within the voxel, with an overall signal distortion and amplitude reduction.^22^ This phenomenon is independent of whether a standard or DQ‐filtered acquisition is used. It is exacerbated when using the MRI body coil for signal excitation, compared with smaller RF transmit coils, which locally can achieve higher RF fields. Thus, while lactate can be detected using standard PRESS sequences, the results will be unreliable and a high‐bandwidth acquisition sequence such as fLASER is required for correct measurement of lactate concentration.

In general, glioblastomas tend to have high lactate concentrations, but with a large variability (e.g. 11.7 ± 7.0 mM; Reference 2). In the present study lactate was measureable in only 54% of the tumours. The fLASER sequence intrinsically has better sensitivity for the detection of lactate than the standard PRESS sequence (owing to its minimal chemical shift displacement artefact^18^). Although the sensitivity of these acquisitions was slightly reduced by the relatively short *T*_R_ used (as mentioned above), the low levels of lactate in 45% of the spectra are likely to be largely attributable to the heterogeneous population of patients studied at different stages of treatment, and in some cases to partial volume effects. The slightly higher water linewidths in spectra similar to that of Figure 1C (those in which lactate was only detected using the DQ filter) showed that poor shimming was not primarily responsible for absence of lactate signals.

Glioblastomas and brain metastases tend to have very high lipid concentrations compared with normal brain and with other brain tumours.^2^ They arise from cytoplasmic lipid droplets.^41^ They are often the result of a stress response, and may play a role in detoxification of the cell, or act as an alternative energy source. They tend to increase following radiation therapy, reflecting macrophage activation and the production of necrosis.^42^

While signals from lipids are largely eliminated by the DQ filter, signals from other metabolites with coupled spins will be partially retained. In particular, signals from threonine, which also has a doublet at 1.3 ppm, are likely to be effectively co‐edited. In normal brain the concentration of threonine is reported to be similar to that of lactate (approximately 0.3 and 0.4 mmol/kg wet weight respectively^43^). In tumour, concentrations of threonine have been reported to be not greatly different from those in normal brain,^44^ but with a twofold increase between Grade 2 and Grade 4 glioma.^45^ Since tumour concentrations of lactate are between about 1.5 and 14 mM,^2^ it is most likely that DQ‐filtered signals at 1.3 ppm arise primarily from lactate. However, the possibility that significant contributions may originate from threonine cannot be excluded. Signals from lactate and threonine can be distinguished in the brain, for example using l‐COSY methods^46^ and *J*‐difference editing,^47^ but these methods take longer to acquire and are not single‐shot methods, and so are not readily transferrable to tissues experiencing motion.

In addition to the possible presence of threonine, most DQ‐filtered spectra show evidence of other metabolites being partially passed by the DQ filter. In this study 13 data sets were omitted from the analysis as the DQ‐filtered spectra were observed to include unidentified peaks that might have potentially interfered with measurement of the lactate resonance. The unidentified peaks may have arisen from other metabolites or components of the macromolecular baseline that were partially passed by the filter. It is possible that some may have also been caused by an artefact such as out‐of‐volume ghosting.^26^ One standard acquisition (non‐filtered) spectrum demonstrated this artefact and was discarded. We have subsequently confirmed that this artefact can be eliminated by doubling the length of the spoiler gradients. Many lactate signals were on the borderline of detection, and either a larger voxel or longer acquisition is really required for unambiguous measurement in these cases. This is a definite limitation of the method.

In conclusion, the fLASER DQ filtering sequence has been demonstrated in a clinical setting. While the standard acquisition method can resolve lactate under conditions of good magnetic field homogeneity and small lipid content, the DQ filter was essential for estimating lactate concentrations in the presence of significant amounts of lipid. In the absence of lipid the method has been found to yield concentrations consistent with those measured using the standard acquisition methods. This single‐shot approach will be especially useful for measuring lactate in tissues experiencing motion.

## Abbreviations used

CRLB: Cramér–Rao lower bound
DQ: double quantum
fLASER: LASER using FOCI pulses
FOCI: frequency offset corrected inversion
LASER: localization by adiabatic selective refocusing
LW_water_: water linewidth
